# Conservative Management of Idiopathic Spontaneous Intraperitoneal Hemorrhage

**DOI:** 10.7759/cureus.96914

**Published:** 2025-11-15

**Authors:** Christian John S Capirig, Shunsuke Kondo, Blake Kadomoto, Joshua J Garcia

**Affiliations:** 1 Internal Medicine, University of Hawaii John A. Burns School of Medicine, Honolulu, USA

**Keywords:** abdominal apoplexy, case report, conservative management, hemoperitoneum, idiopathic spontaneous intraperitoneal hemorrhage

## Abstract

Idiopathic spontaneous intraperitoneal hemorrhage (ISIH), also known as abdominal apoplexy, is a rare and life-threatening cause of acute abdomen that typically requires surgical or endovascular intervention. Conservative treatment is rarely reported, particularly in the setting of hemorrhagic shock. We describe a 59-year-old postmenopausal woman with no anticoagulant use who initially presented with nonspecific gastrointestinal symptoms and then developed hypotension, lactic acidosis, and a significant drop in hemoglobin. Computed tomography (CT) angiography revealed a large mesenteric hematoma with moderate hemoperitoneum and a small focus concerning for extravasation. Follow-up imaging showed no active bleeding, and the patient remained without peritoneal signs. She was managed conservatively in the intensive care unit with fluid resuscitation, brief vasopressor support, and blood transfusion. Her condition stabilized, and she was discharged within a week. Repeat imaging at three months showed complete resolution. This case demonstrates that in carefully selected patients with ISIH, conservative management can be safe and effective. Key considerations include hemodynamic stabilization after resuscitation, absence of peritonitis, no active bleeding on repeat imaging, and availability of close inpatient monitoring. A multidisciplinary approach may support non-operative treatment in situations where surgical or endovascular intervention is not immediately necessary.

## Introduction

Idiopathic spontaneous intraperitoneal hemorrhage (ISIH), also known as intra-abdominal apoplexy, is an extremely rare and potentially life-threatening condition. Its global prevalence remains unknown, and it has been documented in only a limited number of case reports. ISIH occurs when blood vessels in the abdomen, most commonly splanchnic vessels, visceral arteries (such as the middle colic artery), or, in rare instances, other smaller abdominal vessels, spontaneously rupture [1]. Although the exact cause remains unclear, ISIH is typically observed in males, being two to three times more likely than in females, and is often associated with conditions like advanced liver disease, atherosclerosis, and hypertension [2]. However, there are also reports of this condition occurring in younger adults and even adolescents. The clinical presentation of ISIH can be highly variable. Clinicians should maintain a high level of suspicion in patients who present with vague epigastric symptoms, such as nausea, vomiting, and nonspecific abdominal pain, followed by the sudden onset of acute abdominal distress and hemodynamic instability. While computed tomography (CT) angiography is considered the most effective imaging modality for confirming the diagnosis, other diagnostic tools, such as ultrasound, magnetic resonance imaging (MRI), and direct intraoperative visualization, have also been employed in some cases [2,3]. Most patients require surgical intervention, as conservative management in hemodynamically unstable individuals is exceedingly rare. In this report, we present a unique case of successful non-operative management in a patient with hemorrhagic shock due to ISIH, highlighting the potential for conservative treatment in carefully selected cases.

## Case presentation

This is a 59-year-old Asian postmenopausal woman who presented to the emergency department with acute-onset dizziness while walking. She described the sensation as vertigo, lasting several hours and associated with nausea and non-bloody, non-bilious vomiting. She denied headache, head trauma, abdominal pain, bowel changes, dysphagia, dysarthria, diplopia, melena, hematochezia, abdominal trauma, or uncontrolled hypertension. Her past medical history included emphysema, osteoporosis, hypercholesterolemia, depression, and coronary artery disease. She was a former smoker with an eight-pack-year history and denied alcohol or illicit drug use. Her home medications included citalopram, alendronate, ascorbic acid, and calcium carbonate. She was not on any anticoagulant or antiplatelet therapy. A colonoscopy performed five years prior was unremarkable.

On initial evaluation, she was alert and oriented, in mild distress from nausea. Her vital signs were within normal limits: blood pressure was 122/76 mmHg, heart rate 88 beats per minute, respiratory rate 16 breaths per minute, oxygen saturation 98% on room air, and temperature 36.9°C. Physical examination revealed a well-perfused but slightly diaphoretic patient. Cardiopulmonary examination was unremarkable with clear breath sounds and normal heart tones. Her abdomen was soft, non-distended, and non-tender, with normoactive bowel sounds and no signs of peritoneal irritation. Neurologic exam was non-focal, with intact cranial nerves, normal strength, and no signs of cerebellar dysfunction or gait abnormality. She was managed conservatively with intravenous fluids and diphenhydramine for symptomatic relief.

Approximately three hours after presentation, she acutely decompensated, developing hypotension (blood pressure 78/48 mmHg), tachycardia (heart rate 121 bpm), diaphoresis, and lethargy. She became pale and minimally responsive to verbal stimuli. Repeat physical examination revealed an ill-appearing patient with cool extremities, delayed capillary refill, and weak peripheral pulses. Her abdomen remained soft but was now mildly distended with new mild tenderness in the left upper quadrant. There was no guarding, rebound tenderness, or rigidity. Neurologic examination was limited due to somnolence but showed no focal deficits. Laboratory testing revealed a marked drop in hemoglobin from 11.7 g/dL to 5.9 g/dL within three hours (Table 1). Electrolytes, renal and hepatic panels, and pancreatic enzymes were within normal limits. Arterial blood gas showed severe metabolic acidosis with a lactate level of 12 mEq/L. Coagulation parameters were normal, and electrocardiography showed inferior ST depressions, though serial troponins remained negative (Table 2).

**Table 1:** Serial CBC values during the initial ED course MCV: mean corpuscular volume; MCH: mean corpuscular hemoglobin; MCHC: mean corpuscular hemoglobin concentration; RDW: red cell distribution width; CBC: complete blood count; ED: emergency department

Lab test	Reference range	11/14/2023 20:07 (hour 0)	11/14/2023 23:34 (hour 3)	11/16/2023 (hour 48)
White blood cell count	3.8-10.8×10^3^/uL	9.98×10^3^/uL	10.88×10^3^/uL	14.9×10^3^/uL
Red blood cell count	3.6-5.4×10^6^/uL	4.8×10^6^/uL	2.38×10^6^/uL	3.69×10^6^/uL
Hemoglobin	11.2-15.7 g/dL	11.7 g/dL	5.9 g/dL (repeated 2×)	10.2 g/dL
Hematocrit	34.1-44.9%	36.8%	20.1%	29.5%
MCV	79.4-98.4 fL	76.7 fL	84.5 fL	79.9 fL
MCH	26-34 pg	24.4 pg	24.8 pg	27.6 pg
MCHC	32-36 g/dL	31.8 g/dL	29.4 g/dL	34.6 g/dL
RDW	11.6-14.4%	13.6%	14%	14.2%
Platelet count	151-424×10^3^/uL	311×10^3^/uL	181×10^3^/uL	121×10^3^/uL

**Table 2:** Comprehensive laboratory profile on admission and initial evaluation during hemodynamic decompensation BUN: blood urea nitrogen; SGOT: serum glutamic-oxaloacetic transaminase; AST: aspartate aminotransferase; SGPT: serum glutamic-pyruvic transaminase test; ALT: alanine aminotransferase; eGFR: estimated glomerular filtration rate; PT: prothrombin time; INR: international normalized ratio; PTT: partial thromboplastin time; CK: creatine kinase; CKD-EPI: Chronic Kidney Disease Epidemiology Collaboration; Cr: creatinine

Lab test	Reference range	Result
Glucose	70-99 mg/dL	322 mg/dL (H)
BUN	6-23 mg/dL	15 mg/dL
Creatinine	0.6-1.4 mg/dL	0.8 mg/dL
Sodium	133-145 mEq/L	139 mEq/L
Potassium	3.3-5.1 mEq/L	3.4 mEq/L
Chloride	95-108 mEq/L	103 mEq/L
CO2	21-30 mEq/L	22 mEq/L
Calcium	8.3-10.5 mg/dL	8.9 mg/dL
SGOT (AST)	0-40 IU/L	45 IU/L (H)
SGPT (ALT)	0-41 IU/L	51 IU/L (H)
Alkaline phosphatase	35-129 IU/L	65 IU/L
Bilirubin, total	0-1.2 mg/dL	0.5 mg/dL
Total protein	6.4-8.3 gm/dL	7.1 gm/dL
Albumin	3.5-5.2 gm/dL	4.4 gm/dL
eGFR (CKD-EPI Cr 2021)	≥90 mL/min/1.73 m²	85 mL/min/1.73 m² (L)
Anion gap	14-20 mEq/L	17 mEq/L
PT	N/A	13.9
INR	0.8-1.1	1.1
Activated PTT	N/A	27.4
Lactic acid	0.5-2.2 mEq/L	12.2 mEq/L (H)
CK, Total	26-192 IU/L	87 IU/L
Troponin T Gen 5	<19 ng/L	<6 ng/L
Troponin delta	N/A	0
Magnesium	1.6-2.6 mg/dL	1.7 mg/dL
Lipase	13-60 U/L	24 U/L

**Figure 1:**
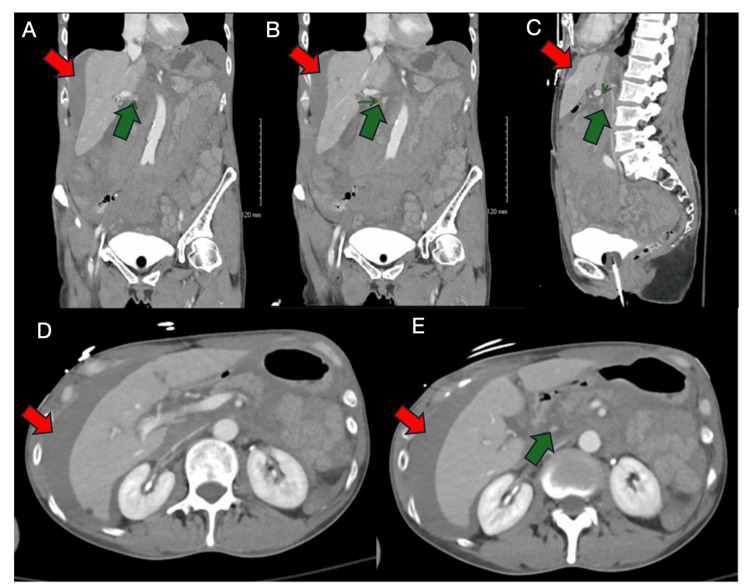
Initial multiplanar CT imaging on day 0 (axial, coronal, and sagittal views). CT images demonstrate a large mesenteric mass suspicious for hematoma, with differential considerations including lymphadenopathy or lymphoma (A) Coronal view (anterior): moderate hemoperitoneum (red arrow) with a small focus of contrast extravasation (green arrow) in RUQ. (B) Coronal view (posterior): moderate hemoperitoneum (red arrow) with a small focus of contrast extravasation (green arrow) in RUQ. (C) Sagittal view: hemoperitoneum (red arrow) with multiple foci of contrast extravasation (green arrow) in RUQ. (D) Axial view (superior): moderate hemoperitoneum (red arrow) without a definite extravasation focus. (E) Axial view (inferior): hemoperitoneum with a small focus of contrast extravasation in RUQ. CT: computed tomography; RUQ: right upper quadrant

**Figure 2:**
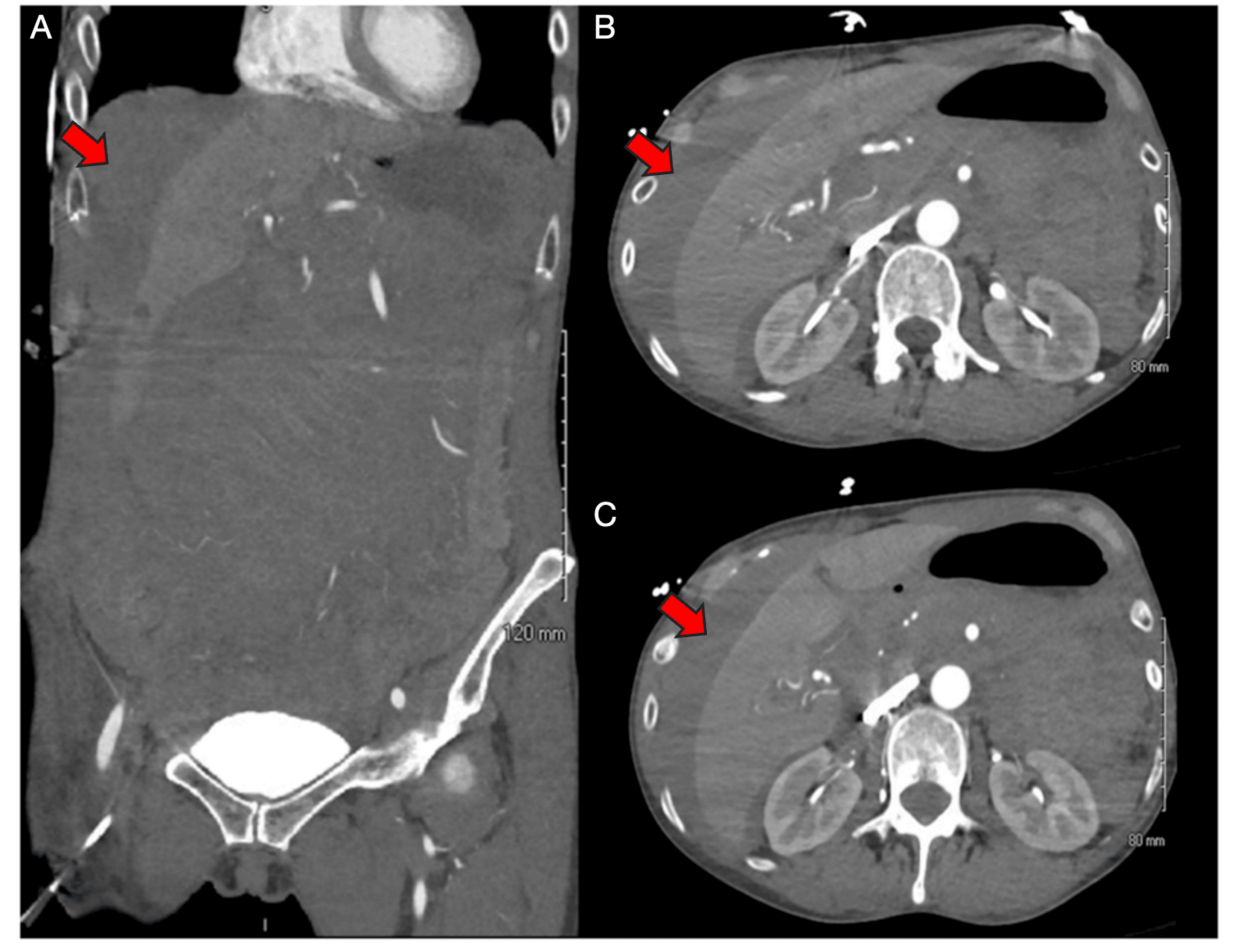
CTA of the abdomen and pelvis (two hours after the initial CT of the abdomen and pelvis). Repeat CTA of the abdomen and pelvis performed approximately two hours after the initial study shows no definite source of active contrast extravasation. The previously described small focus of contrast extravasation is less well delineated. Aorta and major abdominal vessels (celiac trunk, SMA, renal arteries) appear normal without stenosis. There is no evidence of aortic injury, GI bleeding, or new pathology. The IVC remains flattened, and the right femoral central line is unchanged. Overall, there is no interval change in the moderate hemoperitoneum, and no clear source of ongoing bleeding was identified (A) Coronal view: moderate hemoperitoneum (red arrow); no definite active contrast extravasation identified. (B) Axial view (superior): moderate hemoperitoneum (red arrow); no definite source of bleeding; abdominal vessels appear normal. (C) Axial view (inferior): stable hemoperitoneum (red arrow); no active extravasation or new pathology. CTA: computed tomography angiogram; CT: computed tomography; SMA: superior mesenteric artery; GI: gastrointestinal; IVC: inferior vena cava

An urgent contrast-enhanced CT angiogram (CTA) of the abdomen and pelvis revealed a large mesenteric hematoma with moderate hemoperitoneum and a small focus of contrast extravasation in the right upper abdomen concerning for active bleeding (Figure 1). She was transferred to the surgical intensive care unit (ICU) and resuscitated with multiple intravenous fluid boluses, vasopressor support (norepinephrine and vasopressin), and transfusion of three units of packed red blood cells. Despite the need for pressors, she remained alert and oriented and reported only mild abdominal discomfort. Serial abdominal exams throughout the ICU stay were benign, with no evidence of peritoneal signs such as guarding, rebound, or rigidity. A repeat CTA performed two hours later showed no definitive source of active bleeding; the previously visualized extravasation was less well delineated, and the aorta and major visceral arteries were normal without stenosis or dissection (Figure 2). The inferior vena cava appeared flattened, and no new abnormalities were noted. A multidisciplinary team, including surgery and interventional radiology, decided to continue non-operative management given her stabilizing hemodynamics, declining vasopressor requirements, preserved mental status, and resolving lactic acidosis. Interventional radiology was consulted, but no embolization target was identified.

**Figure 3:**
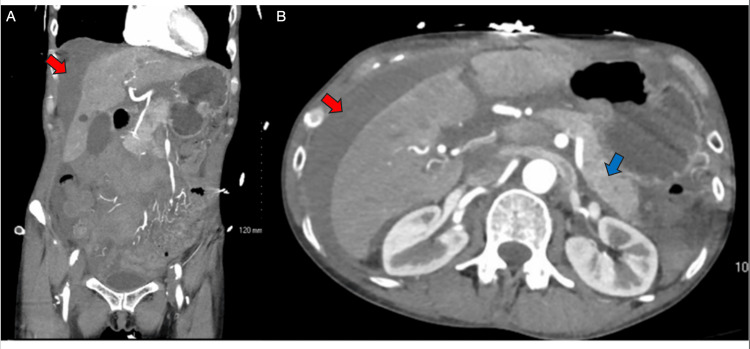
Day 2 repeat CTA of the abdomen and pelvis (multiplanar views). Repeat CTA on day 2 shows stable mesenteric hematoma with faint hyperattenuation in the right upper abdomen, likely due to hyperemia or resolving extravasation. No new active bleeding is identified. Duodenal and jejunal wall thickening is noted, suggestive of enteritis (A) Coronal view: stable mesenteric hematoma with faint hyperattenuation in RUQ (red arrow); no new extravasation. (B) Axial view: stable hematoma (red arrow) with duodenal and jejunal wall thickening (blue arrow), suggestive of enteritis. CTA: computed tomography angiogram; RUQ: right upper quadrant

On hospital day 2, a third CTA demonstrated a stable mesenteric hematoma with persistent faint hyperattenuation in the right upper abdomen, likely related to resolving extravasation or hyperemia. There was also new mild wall thickening of the proximal duodenum and jejunum, suggestive of enteritis (Figure 3). By this time, her hemodynamics had normalized, and her hemoglobin had improved to 10.2 g/dL (Table 1). She remained afebrile, her abdominal exam continued to show no peritoneal signs, and her vasopressors had been discontinued. She was treated with intravenous piperacillin-tazobactam and vancomycin empirically, along with analgesia and supportive care.

**Figure 4:**
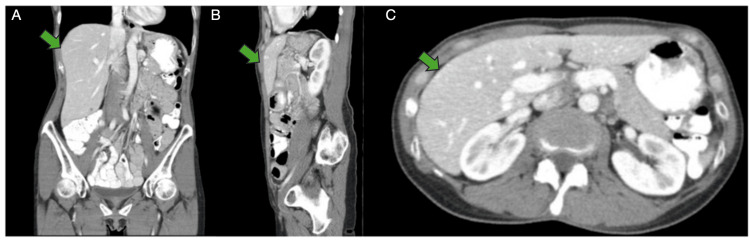
Repeat CT scan after three months in outpatient. Complete resolution of hemoperitoneum (A) Coronal view: complete resolution of hemoperitoneum (green arrow); normal abdominal viscera. (B) Sagittal view: no residual hematoma or active bleeding (green arrow); stable appearance. (C) Axial view: resolution of prior hemoperitoneum (green arrow) with normal solid organs and vasculature. CT: computed tomography

**Figure 5:**
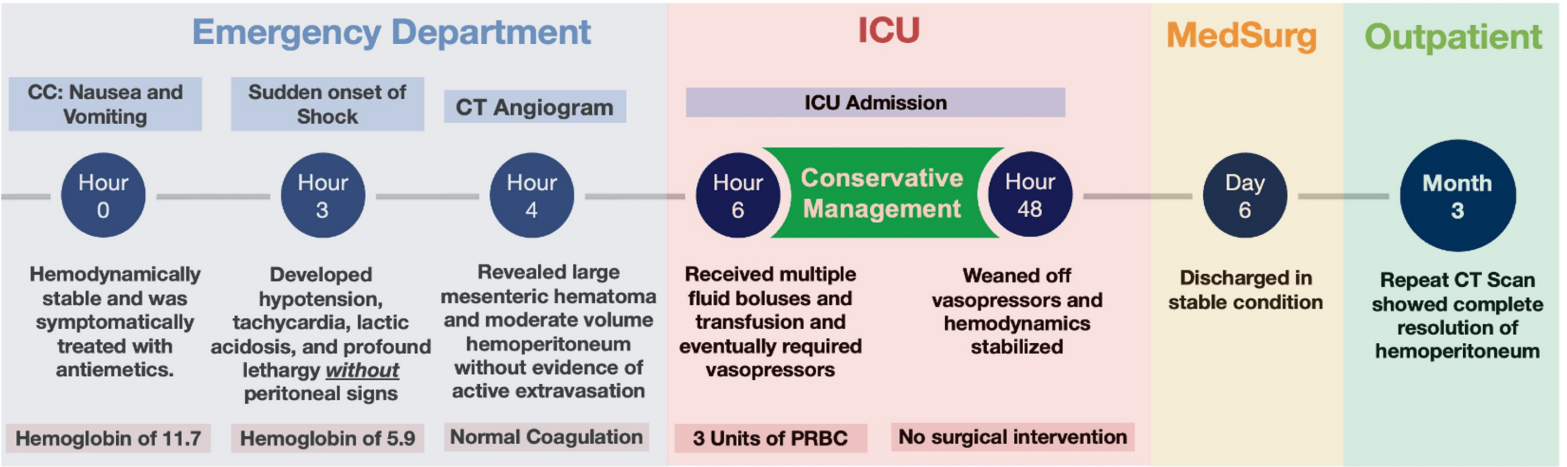
Timeline of non-operative management in idiopathic spontaneous intraperitoneal hemorrhage. The patient presented with nausea and vomiting and later developed hemorrhagic shock. CT imaging revealed a large mesenteric hematoma with moderate hemoperitoneum but no active bleeding. Managed conservatively in the ICU with transfusions and vasopressors, the patient stabilized without surgical intervention and was discharged on day 6. Follow-up imaging at three months confirmed complete resolution CT: computed tomography; ICU: intensive care unit; PRBC: packed red blood cells

The patient was discharged in stable condition on hospital day 6 with resolution of symptoms. At the outpatient follow-up, the patient remained hemodynamically stable. Physical examination was unremarkable: she was alert and oriented, vitals were within normal limits, her abdomen was soft and non-tender with no distension or palpable masses, and there were no signs of recurrent bleeding or infection. A repeat CT scan at three months confirmed the complete resolution of the hemoperitoneum (Figure 4). A summary of the clinical and radiographic events is shown in Figure 5. 

## Discussion

ISIH, also known as abdominal apoplexy, refers to non-traumatic bleeding from intra-abdominal vessels without an identifiable underlying cause such as aneurysm, trauma, or coagulopathy. First described in the early 20th century [4], ISIH remains a rare clinical entity with fewer than 200 cases reported to date [5]. It typically presents in middle-aged to elderly males and is most commonly associated with predisposing conditions such as hypertension, atherosclerosis, or advanced liver disease [6]. Clinical manifestations are often nonspecific, ranging from vague abdominal discomfort to signs of hypovolemic shock, making diagnosis challenging. In most cases, exploratory laparotomy is performed both to confirm the diagnosis and to achieve hemostasis, particularly in unstable patients. Physical examination findings in ISIH are often nonspecific but typically include acute abdominal pain that can range from mild to severe, sometimes accompanied by vomiting and anorexia. On examination, patients may exhibit abdominal tenderness, rebound pain, and abdominal rigidity or peritonism, reflecting peritoneal irritation due to blood in the peritoneal cavity [2,7]. In some cases, abdominal distension is noted. As bleeding progresses, signs of hemodynamic instability such as tachycardia and hypotension may develop, and in severe cases, patients can present in hypovolemic shock with pallor and altered consciousness [7,8]. The clinical course may involve an initial phase of pain, a latent period with fewer symptoms, and a terminal phase with worsening shock if bleeding continues [2,8]. CT imaging remains the primary modality for evaluating ISIH, especially in the acute setting [9]. Table 3 summarizes the imaging findings of ISIH [9,10]. On unenhanced CT, hemoperitoneum is typically seen as hyperattenuating free fluid within the peritoneal cavity, often measuring 30-45 Hounsfield units (HU). A particularly important feature is the sentinel clot sign, referring to a localized hyperdense area (>60 HU) of clotted blood that tends to form adjacent to the bleeding site, offering a clue for potential source localization even when a causative lesion is not visualized. Attenuation can vary widely (20-90 HU) depending on the age of the bleed and is often heterogeneous due to intermittent bleeding or partial clot resorption [10]. Advanced CT technologies, including multidetector-row helical CT, allow for improved detection of small volumes of hemorrhage and subtle contrast extravasation. In some cases, active extravasation appears as serpiginous or nodular foci nearly isodense to adjacent vessels on contrast-enhanced phases. Additional signs include the hematocrit effect, where layering of red blood cells creates a fluid-fluid level, and organized hematomas in sites such as the omental bursa, pelvis, or paracolic gutters. The lack of solid organ injury, mass, aneurysm, or iatrogenic cause supports the diagnosis of ISIH, which is ultimately one of exclusion.

**Table 3:** Imaging findings in idiopathic spontaneous intraperitoneal hemorrhage CT: computed tomography; HU: Hounsfield units Table created independently by authors using information from [9,10]

Imaging finding	Description	Diagnostic implication
Hyperattenuating peritoneal fluid	Fluid measuring 30-45 HU on unenhanced CT (may range from 20 to 90 HU)	Indicates acute hemoperitoneum
Sentinel clot sign	Localized clotted blood with >60 HU near the bleeding site	May help approximate the hemorrhage source
No visible source	Absence of aneurysm, mass, trauma, or visceral injury	Suggests idiopathic etiology
Active extravasation	Hyperdense foci isodense to vessels on contrast-enhanced CT (if present)	Sign of ongoing bleeding; may require embolization or surgery
Hematocrit effect	Fluid-fluid layering with a dependent high-attenuation layer due to the sedimentation of erythrocytes	Suggests recent, possibly ongoing hemorrhage
Heterogeneous or loculated fluid	Mixed-attenuation blood with nodular or linear high-density areas	Reflects intermittent bleeding or clot organization
Organ displacement or mass effect	May be seen with large-volume hemorrhage or omental hematomas	Can mimic tumor; careful assessment needed
No enhancement of adjacent tissue	Excludes enhancing mass or vascular anomaly	Supports non-tumor, non-aneurysmal cause

Despite the growing body of case reports on ISIH, several aspects remain poorly understood. Notably, most published cases involve patients with identifiable risk factors, such as vascular malformations, pseudoaneurysms, or advanced liver disease, and typically necessitate surgical intervention due to hemodynamic instability or peritoneal signs [8,11]. In contrast, the optimal management strategy for patients presenting with hemorrhagic shock in the absence of peritonitis or overt imaging evidence of active bleeding is unclear. While conservative treatment has been described in hemodynamically stable patients, there is limited evidence supporting its use in hemodynamically unstable individuals [5,12]. This report contributes to the limited literature by describing a case of ISIH managed conservatively in a patient who initially presented in shock but stabilized without surgical or interventional radiologic procedures. Clarifying the clinical features that may support such a non-operative approach could refine risk stratification and management algorithms for this rare condition. This case underscores that conservative management can be a viable strategy in selected ISIH patients. However, our patient demonstrates that, even in the setting of initial shock, a non-operative approach may be pursued when key stabilizing features are present. These include (1) responsiveness to fluid resuscitation and transfusion without ongoing hypotension, (2) absence of peritoneal signs, (3) lack of active extravasation on follow-up imaging, (4) stable or rising hemoglobin after transfusion, and (5) availability of close ICU monitoring to detect deterioration. In this case, the patient received three units of packed red blood cells and transient vasopressor support and was maintained on serial physical exams and interval CT scans, all of which supported continued conservative care. Multidisciplinary coordination played a pivotal role in the favorable outcome in this case. Surgical, critical care, and interventional radiology teams collaborated to monitor the patient's trajectory and reassess management in real time. The absence of a clear embolization target, improving hemodynamics, and stable imaging findings helped avoid unnecessary operative risks in a patient with significant comorbidities, including emphysema and coronary artery disease. This report contributes to the limited but growing evidence supporting conservative treatment for ISIH in select patients. While urgent surgical intervention remains the mainstay for unstable individuals with ongoing bleeding or peritoneal signs, our case supports that a tailored, vigilant, and multidisciplinary approach may safely achieve favorable outcomes in cases lacking active bleeding and signs of abdominal catastrophe [5,12].

## Conclusions

This case highlights that ISIH, though rare and often life-threatening, can be successfully managed without surgery in carefully selected patients. While operative or endovascular intervention remains the standard of care for unstable patients or those with ongoing bleeding, our patient demonstrates that conservative management may be safe when key conditions are met: hemodynamic stabilization after resuscitation, absence of peritoneal signs, lack of active bleeding on repeat imaging, and the availability of close ICU monitoring. Multidisciplinary decision-making was central to achieving a favorable outcome. This report adds to the limited literature supporting non-operative management and suggests that individualized, vigilant approaches may help avoid unnecessary procedural risks in patients with significant comorbidities. Further accumulation of similar cases will be essential to better define patient selection criteria and refine management algorithms for this rare clinical entity.
